# High-harmonic spectroscopy of impulsively aligned 1,3-cyclohexadiene: Signatures of attosecond charge migration

**DOI:** 10.1063/4.0000227

**Published:** 2024-02-29

**Authors:** Andres Tehlar, Jakob T. Casanova, Andrey Dnestryan, Frank Jensen, Lars Bojer Madsen, Oleg I. Tolstikhin, Hans Jakob Wörner

**Affiliations:** 1 Laboratory of Physical Chemistry, ETH Zürich, 8093 Zürich, Switzerland; 2 Moscow Institute of Physics and Technology, Dolgoprudny 141700, Russia; 3 National Research University Higher School of Economics, Moscow 101000, Russia; 4 Department of Chemistry, Aarhus University, 8000 Aarhus C, Denmark; 5 Department of Physics and Astronomy, Aarhus University, 8000 Aarhus C, Denmark

## Abstract

High-harmonic spectroscopy is an all-optical technique with inherent attosecond temporal resolution that has been successfully employed to reconstruct charge migration, electron-tunneling dynamics, and conical-intersection dynamics. Here, we demonstrate the extension of two key components of high-harmonic spectroscopy, i.e., impulsive alignment and measurements with multiple driving wavelengths to 1,3-cyclohexadiene and benzene. In the case of 1,3-cyclohexadiene, we find that the temporal sequence of maximal and minimal emitted high-harmonic intensities as a function of the delay between the alignment and probe pulses inverts between 25 and 30 eV and again between 35 and 40 eV when an 800-nm driver is used, but no inversions are observed with a 1420-nm driver. This observation is explained by the wavelength-dependent interference of emission from multiple molecular orbitals (HOMO to HOMO-3), as demonstrated by calculations based on the weak-field asymptotic theory and accurate photorecombination matrix elements. These results indicate that attosecond charge migration takes place in the 1,3-cyclohexadiene cation and can potentially be reconstructed with the help of additional measurements. Our experiments also demonstrate a pathway toward studying photochemical reactions in the molecular frame of 1,3-cyclohexadiene.

## I. INTRODUCTION

High-harmonic spectroscopy (HHS) is a well-established technique to probe the structure and dynamics of molecules. Most notably, it has been used to reconstruct molecular orbitals,^1^ observe Cooper minima,^2^ multi-electron effects in atoms,^3^ multi-orbital contributions in molecules,^4–6^ image chemical reactions,^7^ and conical-intersection dynamics,^8^ probe electron-tunneling dynamics,^9^ reconstruct charge migration,^10^ probe molecular chirality,^11,12^ chiral dynamics,^13^ etc., see also Ref. 14 for a review. Notably, HHS has also been extended to solids^15–17^ and liquids.^18,19^ Most gas-phase work has so far been performed on relatively small molecules. Here, we explore the extension of the techniques of HHS to larger, organic molecules. Although related work has been reported in Ref. 20, the two key components toward reconstructing attosecond charge migration in molecules, i.e., molecular alignment/orientation and the comparison of multiple probing wavelengths,^10^ have not been reported for organic molecules.

In this work, we describe the results of a joint experimental and theoretical investigation of HHS of 1,3-cyclohexadiene (CHD) and benzene. These two molecules were chosen because they are structurally closely related: CHD only differs from the benzene molecule by the addition of two hydrogen atoms, which breaks the aromaticity of the benzene ring and makes the molecular structure non-planar (C_2_ symmetry). CHD is, moreover, of particular interest as a consequence of the ring-opening reaction in its ultraviolet-excited state,^21^ which is a promising target for time-resolved HHS,^22^ particularly if the molecules can be impulsively aligned. Whereas benzene is an oblate symmetric-top molecule, CHD is an asymmetric top, but close to the oblate-symmetric-top limit. Here, we demonstrate the impulsive alignment of both CHD and benzene. We probe the rotational wavepackets of both molecules through HHS driven by 800-nm or 1420-nm laser pulses. Comparison of the high-harmonic spectra of CHD emitted by the two driving wavelengths reveals a striking difference. Whereas the emitted intensity follows qualitatively similar modulation patterns as a function of the delay between the alignment and driving pulses for all harmonic orders emitted from the 1420-nm driver, we find two inversions of the modulation patterns at intermediate photon energies, i.e., between 25 and 30 eV and 35 and 40 eV when using an 800-nm driver. Benzene, in contrast, shows no reversal of its intensity modulation when an 800-nm driver is used. To interpret these experimental results, we rely on the previously established combination^10,23^ of accurate strong-field-ionization rates obtained from the weak-field asymptotic theory (WFAT),^24–27^ amplitudes and phases of the recombining photoelectron wave packet from the strong-field approximation (SFA),^28^ and accurate photorecombination matrix elements obtained from ePolyScat.^29,30^ These calculations show that the observed reversal of the intensity modulation in CHD driven by 800-nm pulses can be understood as originating from the interference of high-harmonic emission from multiple orbitals. Specifically, the contributions of the highest four molecular orbitals (HOMO to HOMO-3) were found to be sufficient to explain the observed reversal. This study, thus, experimentally demonstrates the possibility of aligning CHD and benzene and studying the wavelength dependence of their HHS signals. This advance opens the door to studies of charge migration in both molecules and conical-intersection-mediated ring-opening dynamics in CHD.

## II. EXPERIMENTAL METHODS

A commercial titanium/sapphire laser system providing 8-mJ pulses at a repetition rate of 1 kHz and a pulse duration of 27 fs was used. The laser beam was split using beamsplitters, with one part being sent to a delay stage. The other part was either coupled into a commercial optical parametric amplifier to generate 1420-nm (signal) pulses or directly sent to the high-harmonic-generation (HHG) chamber. Additional details on the experimental setup have been given in Ref. 31. The alignment beam, which was used to induce the rotational wave packet, was sent through an optical system consisting of a λ/2 wave plate and two consecutive polarizers placed at Brewster's angle to allow free tuning of the laser intensity without changing other properties. The pump beam was stretched using 2.4 cm of UV fused silica to optimize the alignment of the sample molecules and to reduce the ionized fraction of molecules.

The molecular samples were introduced into the experimental chamber by the use of a pulsed molecular beam created by bubbling helium through a heated container. Due to the low vapor pressure of CHD, the container was heated to 60 °C and the pipe system to the valve to around 85 °C. The vapor pressure of benzene was high enough that no heating was needed. The molecular vapors were entrained in a flow of helium streaming through the container with a backing pressure of 4 bar. We verified that the He carrier gas did not contribute to HHG at the moderate probe intensities used in the present experiments.

The pump and probe beams were focused into the gas jet non-collinearly with a vertical offset of about 2 cm on the common silver-coated focusing mirror (*f* = 50 cm). After observing high-harmonic emission from the probe beam, the gas nozzle was translated along the beam propagation direction to suppress the emission from the long electron trajectories and thereby isolate the emission from the short trajectories. Both beams had the same polarization direction, and their diameters were individually adjusted with irises to optimize the molecular alignment. Optimal overlap between pump and probe was found by first aligning N_2_, because it has a strongly alignment-dependent high-harmonic emission. If the alignment of N_2_ was found to be as good as expected, the sample was switched to one of the organic molecules and reoptimized for maximal alignment. Other than the spatial overlap of the two foci, the optimization parameters were the pump- and probe-beam diameters and intensities, the distance of the interaction region from the orifice of the pulsed valve, as well as the timing and duration of its opening interval.

The emitted high-harmonic radiation was spectrally analyzed with a flat-field imaging spectrometer consisting of a concave grating and a micro-channel plate detector backed with a phosphor screen. The high-harmonic spectra were analyzed by spatially averaging the intensity over the individual high-harmonic orders on the detector.

## III. EXPERIMENTAL RESULTS

**FIG. 1.**
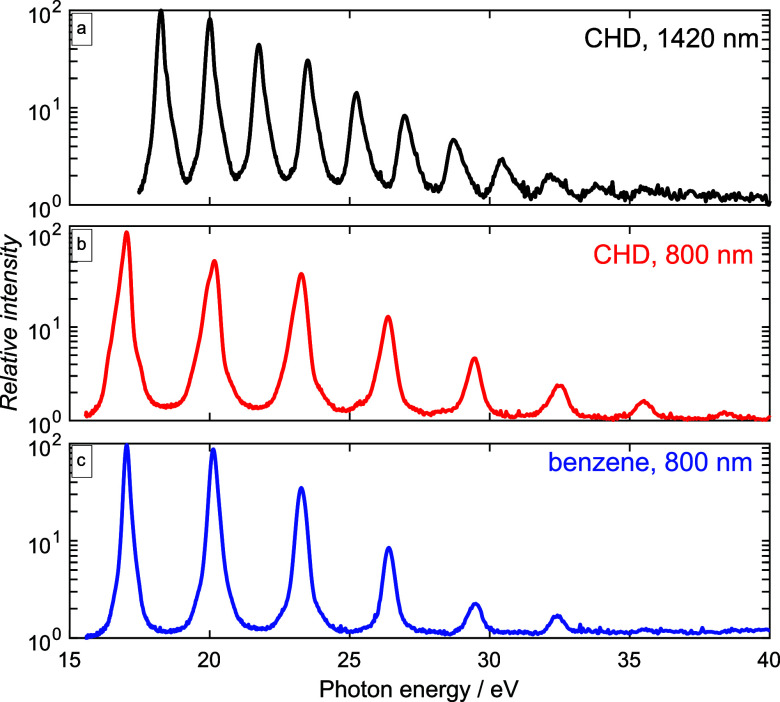
High-harmonic spectra of (a) CHD generated with 40-fs, 1420 nm pulses at a peak intensity of ∼6 $\times 10^{13}$ W/cm^2^, (b) CHD, and (c) benzene generated with 27-fs, 800 nm, ∼1.0 $\times 10^{14}$ W/cm^2^ pulses.

Figure 1 illustrates the measured high-harmonic spectra of CHD probed with 800 or 1420-nm pulses as well as the high-harmonic spectrum of benzene probed with 800-nm pulses for randomly aligned molecules. The pulse durations and peak intensities are indicated in the caption of Fig. 1.

**FIG. 2.**
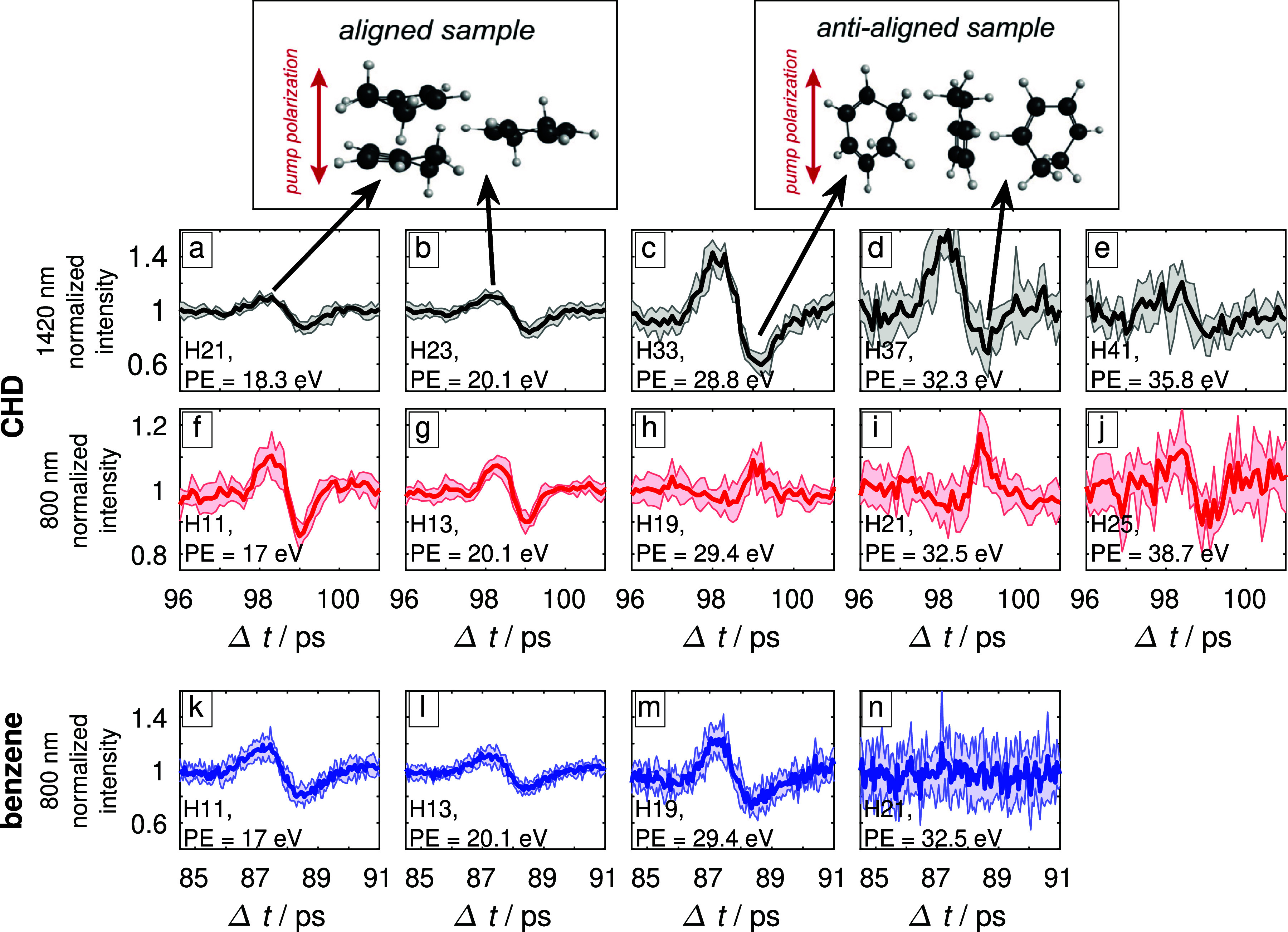
High-harmonic emission of CHD and benzene around the first rotational full revival after impulsive alignment. (a)–(e) The measured high-harmonic emission of CHD at 1420 nm (black) (f)–(j) the measured high-harmonic emission of CHD at 800 nm (red), and (k)–(n) the high-harmonic emission of benzene at 800 nm (blue). The shaded areas mark one standard deviation from the measured mean intensity obtained from 5 (CHD, 1420 nm), 6 (CHD, 800 nm), and 7 (benzene, 800 nm) consecutive scans. PE stands for photon energy. The pulse durations and peak intensities are given in the caption of Fig. 1.

At 800 nm, the spectra of the two molecules are remarkably similar and show only small modulations on top of the general decrease in intensity with increasing harmonic order. Figure 2 shows the baseline-normalized high-harmonic intensity around the full rotational revival at selected photon energies for the different molecules.

Benzene is an oblate symmetric top, and CHD is an asymmetric top that is close to the oblate-symmetric limit. For oblate-symmetric tops, such as benzene, the molecular plane aligns perpendicular to the polarization direction of a linearly polarized alignment pulse.^32^ At the anti-alignment, the molecular plane lies parallel to the polarization direction of the alignment pulse. This situation, which strictly applies to oblate-symmetric tops, is a very good approximation for CHD. The corresponding axis distributions of the CHD molecules at the (anti-)alignment revivals are schematically illustrated in the top panels. The delay of maximal alignment at the first full revival is 98.2 and 87.5 ps for CHD and benzene, respectively. The delay of maximal anti-alignment is 99.0 and 88.4 ps, respectively.

The modulation of high-harmonic emission is significant, typically showing enhanced emission when the molecules are aligned and decreased emission when they are anti-aligned. However, when CHD is probed with 800-nm pulses, the behavior is different, i.e., a first reversal of the intensity modulation is observed around a photon energy of 28 eV and a second reversal around 38 eV. In the case of benzene probed with 800-nm pulses, no such reversals are observed. Instead, all measured harmonic orders display the same type of intensity modulation.

**FIG. 3.**
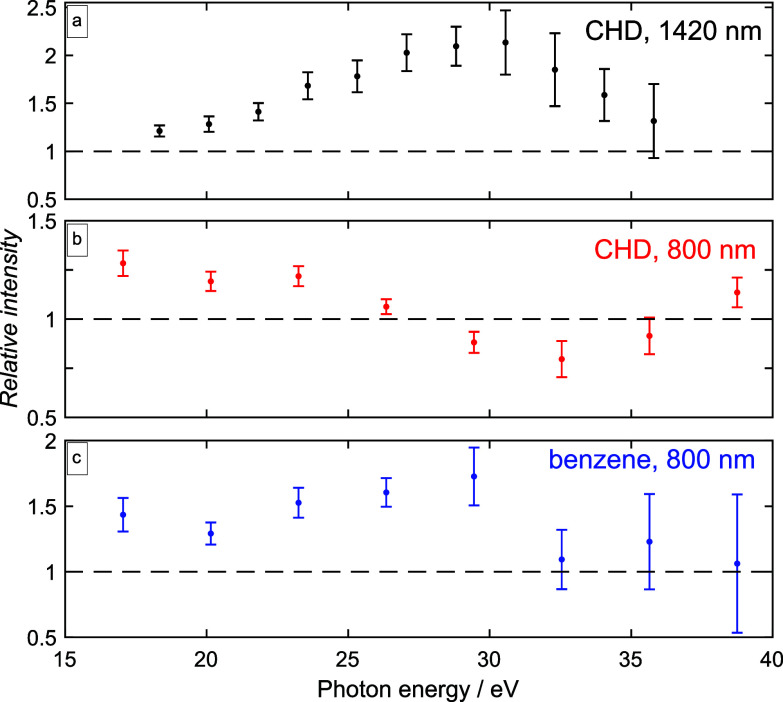
Intensity ratio (aligned over anti-aligned) [see Eq. (1)] determined at the first rotational full revival of the high-harmonic emission of (a) CHD molecules probed with 1420 nm, (b) CHD probed with 800 nm, and (c) benzene probed with 800 nm (blue). The error bars indicate one standard deviation over the measured signals. The pulse durations and peak intensities are given in the caption of Fig. 1.

Figure 3 quantifies this observation in terms of the ratio *r* between the high-harmonic intensities *I* when the molecules are aligned or anti-aligned, which is defined as

$$r(\Omega )=\frac{I(\Delta t_{\text{aligned}},\Omega )}{I(\Delta t_{\text{anti}-\text{aligned}},\Omega )}.$$ (1)

## IV. THEORETICAL RESULTS

We now describe the theoretical model that is used to interpret these experimental results. In the spirit of the three-step model^33^ and based on the successes of previous approaches to modeling high-harmonic spectra,^2,4,5,10,34–36^ we factorize the HHG process into its three steps: tunnel ionization, electron propagation, and recombination. The theoretical methods employed to represent each step are described below. Since we are restricting our considerations to two parallel and linearly polarized (pump and probe) laser pulses, two Euler angles (*θ* and ϕ) suffice to describe the orientation of the molecular axes with respect to the light polarization.

### A. Tunnel ionization

**FIG. 4.**
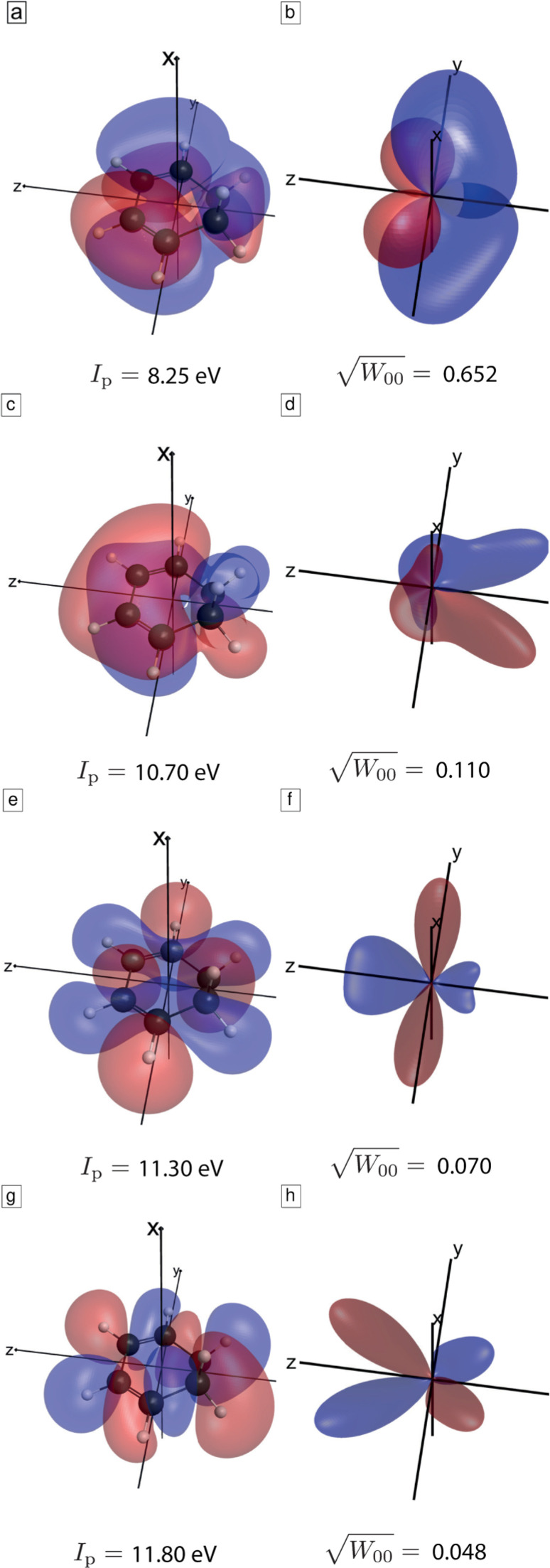
Illustration of the four highest-lying occupied molecular orbitals of CHD and the angle dependence of their WFAT structure factors. Panels (a), (c), (e), and (g) show the orbitals of CHD as isosurfaces of value 0.01 from a Hartree–Fock calculation with the aug-cc-pVQZ basis set. Panels (b), (d), (f), and (h) illustrate the corresponding WFAT structure factors as a three-dimensional plot in which the distance from the origin encodes the magnitude of $G_{00,i}(\theta ,\phi )$ and the color (red or blue) encodes its sign. The square root of the WFAT field factor is given for an intensity of 1 × 10^14^ W/cm^2^.

Whereas the tunnel ionization of atoms is well described by existing theories,^37–39^ the angle dependence of molecular strong-field ionization is still being intensively researched. Popular methods used to describe molecular tunnel ionization are the SFA,^40–43^ the molecular-ADK method,^44^ and the WFAT.^24–27^ Methods that go beyond the single-active-electron approximation include time-dependent density-functional theory,^45–47^ multi-configurational time-dependent Hartree–Fock,^48,49^ and B-spline algebraic diagrammatic construction^50^ methods, to name just a few. Here, we use the WFAT to obtain orientation-dependent total ionization probabilities of molecular orbitals using the polarization consistent quantum-chemistry basis sets^51,52^ to accurately describe the tunneling probability using the integral formalism. Throughout this work, we label the different molecular orbitals and the corresponding channels of high-harmonic emission, defined by ionization and recombination to the same orbital, with the index *i*. Following the WFAT, the leading term in the field strength *F* of the tunnel ionization rate is described as (see, e.g., Ref. 24)

$$\Gamma_{\text{ion},i}(\theta ,\phi )=|G_{00,i}(\theta ,\phi ){|}^{2}W_{00,i}(F),$$ (2)

where *G*_00,*i*_ is a structure factor dependent on the ionized molecular orbital and *W*_00,*i*_ is a field factor, which is given by

$$W_{00,i}=\sqrt{\frac{I_{p,i}}{2}}{\left(\frac{8I_{p,i}}{F}\right)}^{\sqrt{\frac{2}{I_{p,i}}}-1}\;\text{exp}\;\left(-\frac{4\sqrt{2}I_{p,i}^{3/2}}{3F}\right),$$ (3)

where $I_{p,i}$ is the field-free ionization potential of the ionized orbital in atomic units and *F* is the electric field strength in atomic units. The effect of the tunnel ionization is, thus, described with the following spectral factor:

$$a_{\text{ion},i}(\theta ,\phi )=\sqrt{\Gamma_{\text{ion},i}(\theta ,\phi )}P_{\text{ion},i}(\theta ,\phi )=G_{00,i}(\theta ,\phi )\sqrt{W_{00,i}}P_{\text{ion},i}(\theta ,\phi ),$$ (4)

where $P_{\text{ion},i}$ is the orientation-dependent global phase factor of the ionized electron wavepacket, which is contained in $G_{00,i}(\theta ,\phi )$, and *θ*, ϕ are the Euler angles describing the orientation of the molecule with respect to a laboratory-fixed axis system. Figure 4 illustrates the angle dependence of the four highest-lying occupied molecular orbitals of CHD. Note that for easier comparison of the molecular orbitals and $G_{00,i}(\theta ,\phi )$, all figures are given in the molecular frame of reference.

### B. Electron propagation

The propagation of the electron wave packet in the continuum under the effect of the driving laser field is described within the SFA.^28^ These calculations return a complex-valued spectral factor $a_{\text{prop},i}(\Omega )$, which represents the amplitude and phase of the recombining photoelectron wavepacket. The factor $a_{\text{prop},i}(\Omega )$ depends on $I_{p,i}$ as well as the intensity, duration, and wavelength of the driving pulse. It is assumed to be independent of the molecular orientation. The relative phases of the factors $a_{\text{prop},i}(\Omega )$ are responsible for constructive or destructive interference between the different channels at different emitted photon energies Ω. The relative amplitudes of $a_{\text{prop},i}(\Omega )$ are of particular relevance in the cutoff region, where they tend to enhance the contributions of lower-lying (more tightly bound) molecular orbitals compared to the HOMO.

The phase of the propagation term can be approximated as follows:

$$\text{Arg}\left(a_{\text{prop},i}(\Omega )\right)\approx -I_{p,i}\tau +cU_{p}\approx -I_{p,i}\tau ,$$ (5)

where *τ* is the round trip time of the electron in the continuum (transit time), the prefactor *c* is small enough compared to *τ* to be neglected when measuring only emission generated by short electron trajectories,^53^ and *U_p_* is the ponderomotive potential. The complete expression for *c* is given in Ref. 53. In the model used here, *τ* is well approximated by the classical model of an electron in a linearly polarized laser field.^33^ In the calculations presented in this work, we have used the approximate phase given by Eq. (5) because this choice avoids the numerical noise and instabilities of the SFA calculations. Where necessary, we have extrapolated *τ* into the cutoff regions of each channel. We have verified that the approximate phase yields results that are fully consistent with the SFA results.

### C. Photorecombination

Electron–ion recombination is the time-reversed process of ionization.^2,34,54^ It can be described through matrix elements obtained from electron-molecule scattering calculations performed with ePolyScat.^29,30^ The corresponding electron-molecule-scattering problem is solved iteratively with the Schwinger variational principle using the occupied molecular orbitals as an input. Within the dipole approximation and assuming linearly polarized light with polarization direction **n**, the photoionization matrix element can be expressed in atomic units as

$$a_{\text{rec},i}(\theta ,\phi ,\Omega )=\left\langle \psi_{f,k}|r\cdot n|\psi_{i}\right\rangle ,$$ (6)

where $\psi_{f,k}$ describes the final molecular state as an ionized molecule with a continuum electron of asymptotic momentum **k**, −r is the dipole operator, and $\Omega =I_{p}+{\left|k\right|}^{2}/2$.

**FIG. 5.**
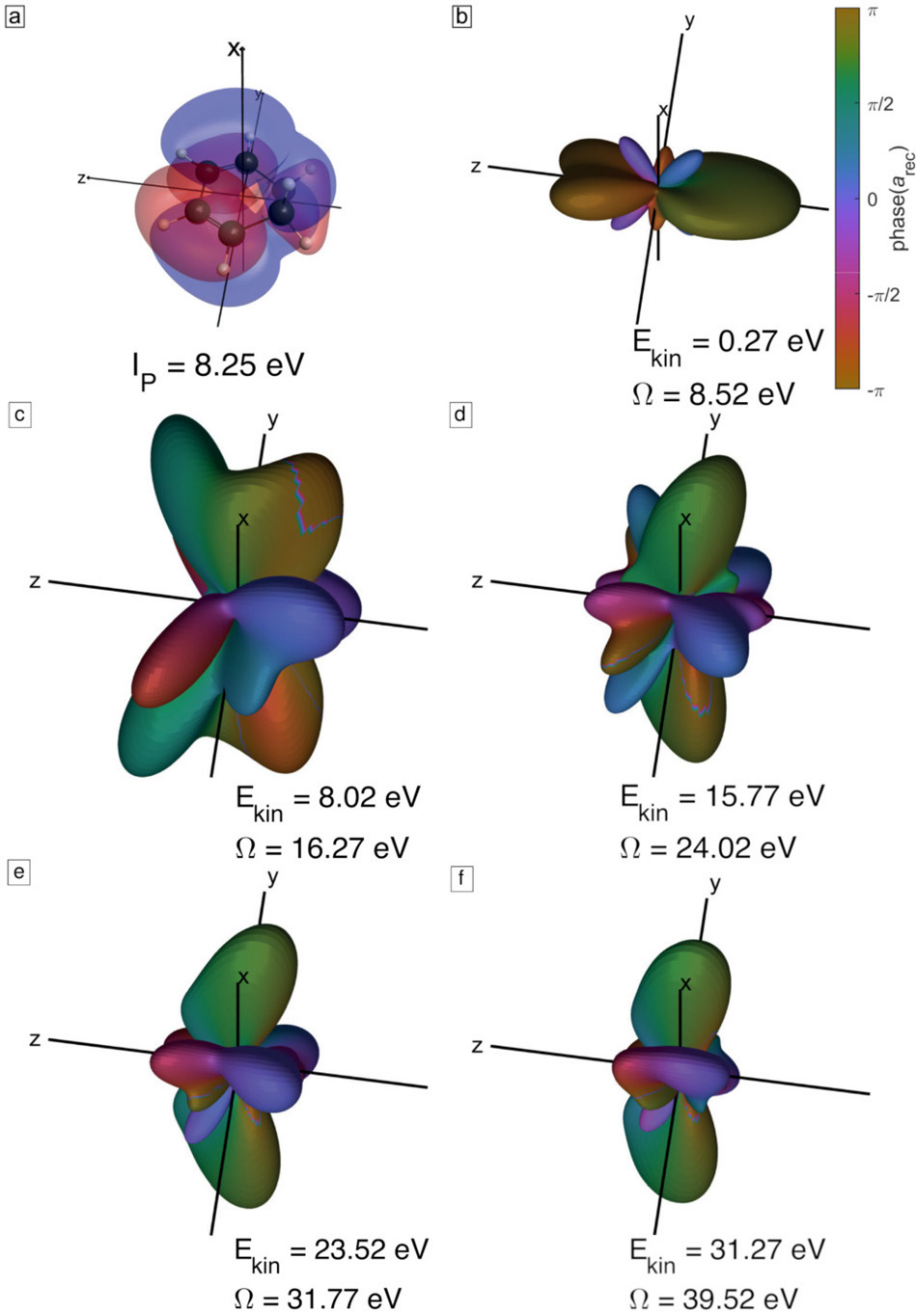
Photorecombination matrix elements to the HOMO of CHD (shown in a) for kinetic energies of (b) 0.27 eV, (c) 8.02 eV, (d) 15.77 eV, (e) 23.52 eV, and (f) 31.27 eV. The corresponding emitted photon energies (Ω) are also indicated. The orbital was obtained from a HF/aug-cc-pVQZ calculation. The photorecombination matrix elements were calculated with ePolyScat.^29,30^ The amplitude is encoded as the distance from the origin, and the phase is color-coded according to the legend in (b).

**FIG. 6.**
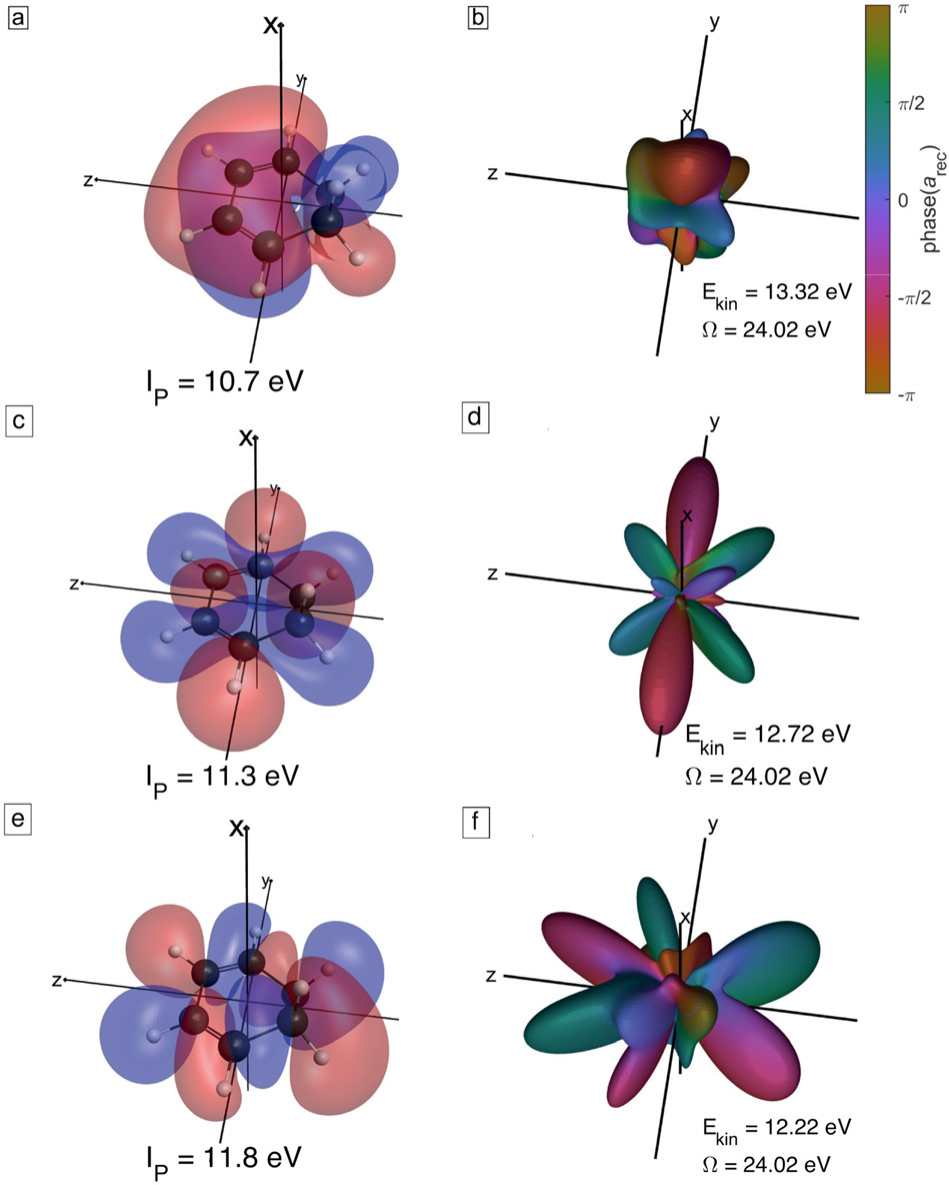
Photorecombination matrix elements to the HOMO-1, HOMO-2, and HOMO-3 of CHD at a fixed photon energy of 24.02 eV, corresponding to different electron kinetic energies. The orbitals are shown in (a), (c), and (e) and the photorecombination matrix elements in (b), (d), and (f). The amplitude of the photorecombination matrix elements is encoded as the distance from the origin and their phase is color-coded according to the legend in (b).

An example of the photorecombination matrix elements to the highest occupied molecular orbital (HOMO) is shown in terms of its amplitude and phase for a range of photon energies in Fig. 5. Whereas the overall angular dependence of the recombination matrix elements reflects the shape of the orbital, the amplitudes and phases are strongly dependent on the kinetic energy of the recombining electron. Especially, for slow electrons, the shape of the recombination matrix elements can vary a lot [Figs. 5(a)–5(d)], whereas smaller variations are observed at higher kinetic energies [Figs. 5(e) and 5(f)]. The structures in the recombination matrix elements can have a significant impact on the high-harmonic spectrum. They are due to (anti-)resonances in the continuum (e.g., Cooper minima^55^ or shape resonances^56^). Because of this, the recombination term cannot easily be predicted and has to be calculated with accurate methods. Especially for very low kinetic energies, the recombination matrix elements show often little similarities with the orbital structure [Figs. 5(a) and 5(b)]. Because of the *I_p_*-dependence of the photoemission energy, the recombination to different orbitals leading to the same photon energy can occur with significantly varying kinetic energy. The recombination of the four different channels leading to an emission at 24.02 eV is shown in Figs. 5(d) and 6.

### D. Calculation of high-harmonic spectra

The electric-dipole moment *d* representing HHG, induced along the polarization direction of the driving field $\hat{\varepsilon }$, is expressed as

$$d(\Omega ,\Delta t)=\hat{\varepsilon }\cdot \vec{d}=\sum_{i}\int_{0}^{2\pi }\int_{0}^{\pi }A(\theta ,\phi ,\Delta t)a_{\text{ion},i}(\theta ,\phi )C_{i}(\Omega )a_{\text{prop},i}(\Omega )a_{\text{rec},i}(\theta ,\phi ,\Omega )\;\text{sin}\;\theta \text{d}\theta \text{d}\phi ,$$ (7)

where $C_{i}(\Omega )$ is the nuclear auto-correlation function that accounts for vibrational dynamics of the cation between ionization and recombination. The only quantity that is sensitive to the pump–probe delay is the axis distribution A(θ,ϕ,Δt). Note that the ansatz of Eq. (7) breaks down when the laser induces transitions between the ionic states during the high-harmonic generation process,^10,57^ which is not included in the present model.

As CHD has two nearly identical rotational constants (*A* = 0.16925 cm^−1^ and *B* = 0.16886 cm^−1^) and *C* = 0.9012 cm^−1^,^58^ it is very well approximated by an oblate symmetric top. The axis distribution A(θ,ϕ,Δt) is defined in the laboratory frame, whereby *θ* is the polar angle between the polarization direction of the laser pulses and the main axis of the alignment distribution given by the direction associated with the extraordinary moment of inertia. In this case, the distribution is isotropic in ϕ, and the axis distribution of aligned molecules can be parametrized by analogy with the case of linear molecules,^59^

$$A_{\text{aligned}}(\theta ,\phi ,\Delta t)=N_{\text{aligned}}\left(1+\alpha /\sqrt{{\;\text{cos}}^{2}(\theta )+\varepsilon^{2}{\;\text{sin}}^{2}(\theta )}\right)$$ (8)

with the parameters *α* and *ε*. They are fixed by the degree of alignment, which is usually given as $\left\langle {\;\text{cos}\;}^{2}(\theta )\right\rangle$ at the time of alignment, and $N_{\text{aligned}}$ is the normalization constant defined by

$$\int_{\phi }\int_{\theta }A_{\text{aligned}}(\theta ,\phi ,\Delta t)\;\text{sin}(\theta )\text{d}\theta \text{d}\phi =1.$$ (9)

The anti-aligned distribution $A_{\text{anti}-\text{aligned}}$ is defined similarly as

$$A_{\text{anti}-\text{aligned}}(\theta ,\phi ,\Delta t)=N_{\text{anti}-\text{aligned}}\left((1+\alpha /\sqrt{{\;\text{cos}}^{2}(\theta +\pi /2)+\varepsilon^{2}{\;\text{sin}}^{2}(\theta +\pi /2)}\right).$$ (10)

Isotropic distributions have an expectation value of $\left\langle {\text{cos}\;}^{2}(\theta )\right\rangle$= 1/3, and anti-alignment expresses itself with a lower value and alignment with a higher value.

The SFA was used to calculate the propagation terms $a_{\text{prop},i}(\Omega )$. Since the phases of the propagation terms presented numerical instabilities, particularly in the cutoff region, their arguments were replaced by Eq. (5). The influence of nuclear motion between tunnel ionization and recombination was included through the nuclear auto-correlation function $C_{i}(\Omega )$, which is obtained through the inverse Fourier transformation of the photoelectron spectrum. This method has been introduced and demonstrated in Refs. 10 and 60. The photoelectron spectrum of CHD was taken from Ref. 61. The photorecombination matrix elements were calculated with ePolyScat using molecular orbitals from a Hartree–Fock calculation with the cc-pVQZ basis set. The HHG calculations presented in this work included the four highest-lying molecular orbitals of CHD with respective (experimental) binding energies of 8.25, 10.7, 11.3, and 11.8 eV.^61^

**FIG. 7.**
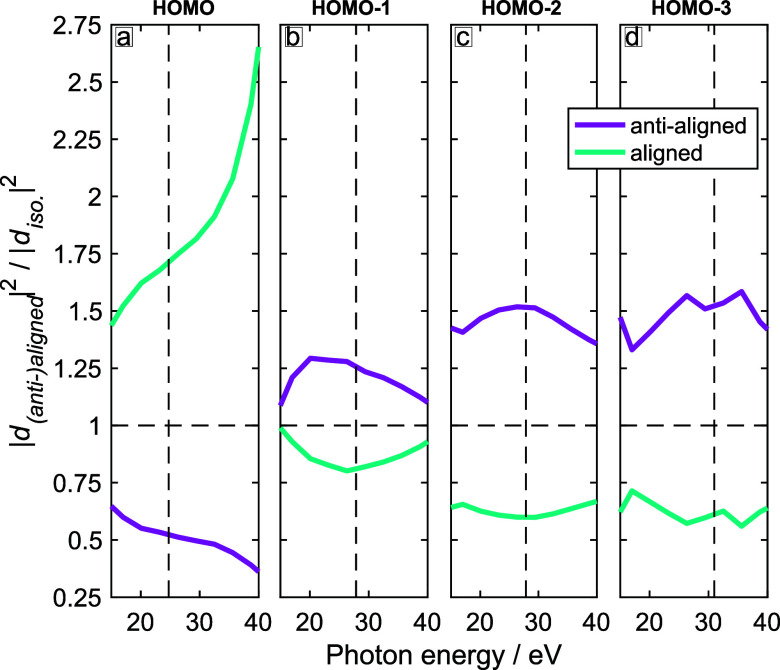
Calculated high-harmonic intensity ratios [(anti-)aligned vs isotropic axis distributions] for CHD at *λ* = 800 nm, a probe intensity of $1.0\times 10^{14}$ W/cm^2^, and an alignment parameter of $\left\langle {\;\text{cos}\;}^{2}(\theta )\right\rangle$ = 0.41. (a) HOMO channel, (b) HOMO-1 channel, (c) HOMO-2 channel, and (d) HOMO-3 channel. The vertical line indicates the photon energy above which the value of *τ* was extrapolated.

**FIG. 8.**
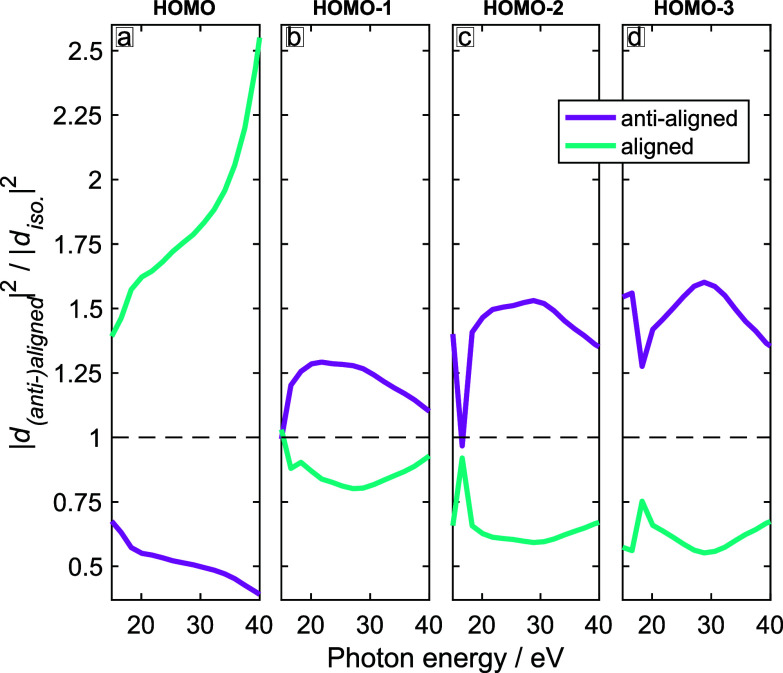
Same as Fig. 7 for *λ* = 1420 nm, $6.0\times 10^{13}$ W/cm^2^ and $\left\langle {\;\text{cos}}^{2}(\theta )\right\rangle$ = 0.41.

Figure 7 shows the channel-resolved ratios of calculated high-harmonic intensities between an aligned and an unaligned (isotropic) sample (cyan lines) or an anti-aligned and an unaligned sample (magenta line). These plots show that alignment enhances the emission from the HOMO and suppresses the emission from HOMO-1 to HOMO-3, whereas anti-alignment has the opposite effect. These effects can be understood from the structures of the four orbitals (Fig. 4) and the corresponding structure factors for strong-field tunneling and photorecombination matrix elements (Figs. 5 and 6). Because the HOMO is an out-of-phase linear combination of the two *π* systems (double bonds), both tunneling and recombination peak at small polar angles relative to the x-axis. Taking the axis distribution of aligned molecules into account, this gives rise to maximal signal when the molecules are aligned, i.e., when the molecular plane is perpendicular to the pump and probe polarizations. The in-phase linear combination of the two *π* systems in the HOMO-1, in contrast, leads to tunneling and photorecombination maximizing at small polar angles relative to the z-axis, which results in the opposite behavior of the intensity ratios, i.e., maximal signal when the molecules are anti-aligned, rather than aligned. The same holds true in the case of the HOMO-2 and the HOMO-3, which are both of $\sigma^{*}$ type. In this case, the structure factors and recombination matrix elements also peak for small polar angles relative to the z-axis, resulting in maximal signal for anti-aligned, rather than aligned molecules. This discussion shows how the structure of these four valence orbitals explains the observed trends of the channel-resolved calculations. Figure 8 shows the same results for a 1420-nm driver. As expected, the wavelength has a limited effect on the intensity ratios because the structure factors and recombination matrix elements are independent of the driving wavelength.

**FIG. 9.**
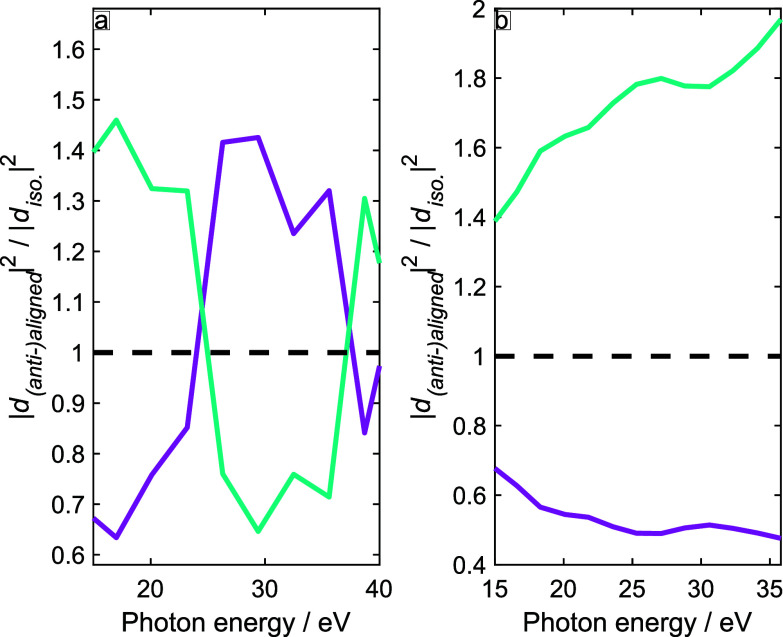
High-harmonic intensity ratios calculated according to Eq. (7), i.e., including all four channels (HOMO to HOMO-3). (a) The results for *λ* = 800 nm, $I=1.0\times 10^{14}$ W/cm^2^ and $\left\langle {\;\text{cos}\;}^{2}(\theta )\right\rangle$ = 0.41. (b) *λ* = 1420 nm, I = $6.0\times 10^{13}$ W/cm^2^, $\left\langle {\;\text{cos}\;}^{2}(\theta )\right\rangle$ = 0.41.

Combining the results for all channels (HOMO to HOMO-3) together, i.e., adding up the complex-valued induced dipole moments, we can predict the observables of the experiment. Figure 9 shows the ratios of calculated high-harmonic intensities between (anti-)aligned and unaligned (isotropic) molecules under the experimental conditions, i.e., for *λ* = 800 nm (panel a) or *λ* = 1420 nm (panel b). Whereas the channel-resolved intensity ratios (shown in Figs. 7 and 8) do not cross the value of one between 20 and 40 eV, the total emission does cross this value twice for *λ* = 800 nm (panel a), but not for *λ* = 1420 nm (panel b).

**FIG. 10.**
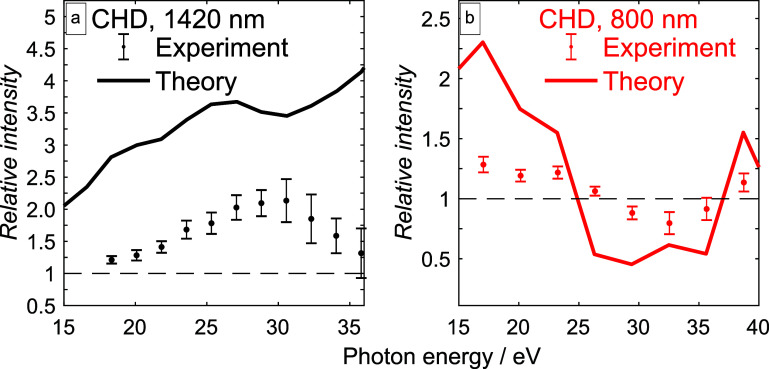
Comparison between the measured and the calculated intensity ratios of CHD (aligned over anti-aligned). The panels (a) and (b) show the same data as (a) and (b) from Fig. 3, also included in the respective subfigure are the calculations. The data shown in (a) are for a driving wavelength of 1420 nm with a peak intensity of $6.0\times 10^{13}$ W/cm^2^ and the data shown in (b) for 800 nm and a peak intensity of $1.0\times 10^{14}$ W/cm^2^. The degree of alignment used in all calculations was $\left\langle {\;\text{cos}\;}^{2}(\theta )\right\rangle$ = 0.41.

With these results at hand, we can now make direct contact with the experimental results, as illustrated in Fig. 10. Panels (a) and (b) show the measured ratios of high-harmonic intensities (aligned over anti-aligned), as determined at the first rotational full revival (Figs. 2 and 3). Panels (c) and (d) show the corresponding calculated ratios. Although the agreement is not quantitative, the contrasting experimental results obtained with the driving wavelengths of *λ* = 1420 nm [panel (a)] and *λ* = 800 nm [panel (b)] are reproduced. The ratios measured and calculated for *λ* = 1420 nm are always larger than one, whereas the ratios obtained for *λ* = 800 nm cross unity twice, once between 25 and 30 eV and once between 35 and 40 eV.

## V. DISCUSSION

These results lead to the interesting question about the origin of the different high-harmonic responses of CHD to different driving wavelengths. As shown in Figs. 7 and 8, the channel-resolved response is qualitatively similar at the two driving wavelengths. Therefore, single-channel effects cannot explain the observed differences. Since the degree of alignment and the peak intensities were identical in the calculations and were kept as similar as possible to the experiments, these effects can also be excluded as the origin of the observed differences. What is left is, therefore, the interference between high-harmonic emission from the different channels. This interference is governed by the relative phases (and amplitudes) of the different channels as given by Eq. (5). The main effect of the driving wavelength is to change the transit time *τ*, which directly scales with the driving wavelength. In other words, the transit times *τ* are longer by the factor 1420/800 between driving wavelengths of 1420 and 800 nm. The relative amplitudes of the propagation terms ( $\left|a_{\text{prop},i}(\Omega )\right|$) also play a role in this interference because the different ionization potentials of the various channels result in different cutoff energies for the emission from these channels, which gives a higher amplitude to the inner-orbital channels in the cutoff region compared to the plateau region (see, e.g., Ref. 31). Overall, we find that the difference in the channel-specific transit times is the main origin of the different calculated and observed high-harmonic responses of CHD at these two driving wavelengths.

These different transit times imply that photorecombination to CHD^+^ occurs over a different range of time intervals elapsed between the ionization and the recombination steps. The range of transit times in the case of an 800-nm driver is 0.7–1.7 fs, and in the case of a 1420-nm driver, it is 1.2–3.0 fs. The sensitivity of the high-harmonic spectra to the driving wavelengths, therefore, shows that the spectra contain information about the relative populations and the relative phases of the electronic states of CHD^+^ at the instant of recombination. If these relative populations and phases could be reconstructed from the experimental data, as demonstrated in Ref. 10, attosecond charge migration in CHD could potentially be reconstructed. What is missing in the present experiments is an explicit measurement of the phase of high-harmonic emission. Such a measurement would be necessary to determine the relative amplitudes and phases of the cationic states.^10^ We note that the dependence of the initial conditions of the electronic wave packet on the parameters of the driving field is not a fundamental obstacle to the reconstruction of charge migration, as demonstrated in Ref. 10.

Another important aspect in reconstructing attosecond charge migration is the entanglement between ion and electron, as discussed in Ref. 14. The reconstruction of charge migration, indeed, necessitates a low degree of entanglement. If and only if the electron–ion system can be written as a direct-product state, is it in principle possible to define the quantum state of the ion. This is only the case in the absence of entanglement. In the presence of weak entanglement, it is still possible to extract a limited amount of information regarding charge migration. The degree of entanglement is defined by the overlap of the continuum wavefunctions between the different channels. This overlap is limited by symmetry, especially in highly symmetric molecules, such as N_2_ or CO_2_, but it can be very high in asymmetric molecules or between ionic states of the same symmetries, such as the two ^2^Π states of iodoacetylene cation, for which attosecond charge migration has been successfully reconstructed.^10^ CHD has C_2_ symmetry, which has the irreducible representations A and B. The symmetries of HOMO to HOMO-3 are A, B, A, and B, in this order. Strong-field ionization from each of these orbitals will populate continuum states of both A and B symmetries, and recombination from both continuum symmetries is dipole-allowed to each of the four ionic states. As a consequence, we find that symmetry does not enforce electron–ion entanglement in high-harmonic spectroscopy of CHD, such that a very low degree of entanglement can be expected, implying that attosecond charge migration can, in principle, be reconstructed.

## VI. CONCLUSION

We have reported the extension of two key techniques of high-harmonic spectroscopy to CHD and benzene, namely, impulsive alignment and measurements with different driving wavelengths. In contrast to benzene, CHD displays two reversals of its alignment modulation under an 800-nm driver, whereas no such inversions are observed with a 1420-nm driver. The first inversion occurs between 25 and 30 eV, and the second occurs between 35 and 40 eV. These results have been reproduced with calculations based on the WFAT, the SFA, and accurate photorecombination matrix elements. These results allow us to conclude that the observed dependence of the high-harmonic response of aligned CHD molecules is the consequence of interference between the emission from different channels, originating from different transit times of the continuum electron. In other words, what we have observed and modeled is the signature of attosecond charge migration in CHD^+^. Reconstructing charge migration in CHD^+^ will require additional phase-sensitive measurements^10^ and/or phase retrieval.^62^ The symmetry analysis of CHD indicates that the degree of electron–ion entanglement can be expected to be very low, such that the reconstruction of charge migration would be meaningful. This opens promising perspectives to reconstructing charge migration in more complex organic molecules than previously demonstrated. Our experiments have, moreover, shown that CHD can be impulsively aligned, which fixes the molecular plane in a well-defined spatial orientation. Combining these one-dimensionally aligned samples with a photoexcitation pulse, e.g., triggering ring-opening of CHD would fix another axis of the molecule and therefore give access to three-dimensionally aligned samples. This opens the perspective of high-harmonic spectroscopy of photochemical reactions in the molecular frame.

## Data Availability

The data that support the findings of this study are available from the corresponding author upon reasonable request.
